# A qualitative study of community elders’ perceptions about the underutilization of formal maternal care and maternal death in rural Nigeria

**DOI:** 10.1186/s12978-019-0831-5

**Published:** 2019-11-11

**Authors:** Arone Wondwossen Fantaye, Friday Okonofua, Lorretta Ntoimo, Sanni Yaya

**Affiliations:** 1 Interdisciplinary School of Health Sciences, University of Ottawa, Ottawa, ON Canada; 2 Women’s Health and Action Research Centre, Benin City, Nigeria; 3 Centre of Excellence in Reproductive Health Innovation (CERHI), University of Benin, Benin City, Nigeria; 4 University of Medical Sciences, Ondo City, Ondo State Nigeria; 5 Federal University Oye-Ekiti, Oye, Ekiti State Nigeria; 6 School of International Development and Global Studies, University of Ottawa, Ottawa, Canada

**Keywords:** Maternal death, Maternal care utilization, Rural, Nigeria, Elders, Community conversations

## Abstract

**Background:**

Underutilization of formal maternal care services and accredited health attendants is a major contributor to the high maternal mortality rates in rural communities in Nigeria. Perceptions of a poor quality of care and inaccessible services in health facilities strongly influence the low use of formal maternal care services. There is therefore a need to understand local perceptions about maternal health services utilization and maternal death. This study thereby aims to explore perceptions and beliefs about the underutilization of formal care and causes of maternal death, as well as to identify potential solutions to improve use and reduce maternal mortality in rural Nigeria.

**Methods:**

Data were collected through 9 community conversations, which were conducted with 158 community elders in 9 rural communities in Edo State, Nigeria. Data from transcripts were analyzed through inductive thematic analysis using NVivo 12 software.

**Results:**

Perceived reasons for the underutilization of formal maternal care included poor qualities of care, physical inaccessibility, financial inaccessibility, and lack of community knowledge. Perceived reasons for maternal death were related to medical causes, maternal healthcare services deficiencies, uptake of native maternal care, and poor community awareness and negligence. Elders identified increased access to adequate maternal care, health promotion and education, community support, and supernatural assistance from a deity as solutions for increasing use of formal maternal care and reducing maternal mortality rates.

**Conclusion:**

Study results revealed that multifaceted approaches that consider community contexts, challenges, and needs are required to develop acceptable, effective and long-lasting positive changes. Interventions aiming to increase use of formal care services and curb maternal mortality rates must target improvements to the technical and interpersonal qualities of care, ease of access, community awareness and knowledge, and allow community members to actively engage in implementation phases.

## Plain English Summary

Nigeria has one of the highest rates of maternal deaths during pregnancy, childbirth and after childbirth in the developing world. The worst rates are seen in rural Nigerian communities. The major contributor to such high rates in rural Nigeria is the underuse of maternal services in health facilities. Instead, many rural Nigerian women use traditional services that are unsafe and not based on scientific evidence. With the traditional influence of community elders’ perceptions and opinions on reproductive health decisions, such as the decision to seek delivery care in a hospital, it is important to understand what they think is causing underuse of health facilities and death during maternity, and to identify potential solutions for their communities.

Informal focus group discussions (community conversations) were conducted with influential community elders in 9 different rural communities in Nigeria. They believed that women underused maternal health services in health facilities because of poor care quality, difficulty of getting to health facilities, high costs of receiving care, and lack of knowledge about maternal health. They believed that medical illnesses, poor availability of certain services, reliance on native maternal care and poor awareness caused maternal deaths. Increasing the accessibility of health facility services, promoting positive health behaviors, community support, and help from God were suggested as solutions for increasing the uptake of health facility services and reducing maternal death rates.

## Background

Accounting for nearly 66% of the global maternal mortality, many sub-Saharan African countries failed to achieve Millennium Development Goal (MDG) 5A of reducing the maternal mortality ratio (MMR) by 75% between 1990 and 2015 [1]. Today, there is a renewed commitment to Sustainable Development Goal (SDG) 3.1 in achieving the target of less than 70 maternal deaths per 100, 000 live births by 2030 [2]. Africa’s most populous country, Nigeria, failed to meet MDG 5A with a percentage change in MMR of only 39.7% between 1990 and 2015 [1]. Recent epidemiological data for Nigeria approximates 58,000 maternal deaths per year, which accounts for the highest absolute number of maternal deaths in the world [1]. Although most maternal deaths are preventable, the inaccessibility and underutilization of formal maternal healthcare services and trained health professionals sustains the high mortality rates across Nigeria and sub-Saharan Africa as a whole [1, 3]. Formal maternal healthcare services refer to evidence-based services provided throughout the continuum of maternal care by accredited health professionals, often in a health facility setting [3]. Less than half of Nigerian women make four or more formal antenatal care visits during their pregnancy, while approximately 60% of childbirths have taken place at home since the 1990s [3]. During the postpartum period, only about 33% of Nigerian women have utilized formal postnatal care since 2003 [3]. Costs of services, distance to health facilities, long waiting times and poor treatment from professional attendants often deter Nigerian women from utilizing formal maternal healthcare services [4].

All parts of the country are affected, but there are major urban-rural disparities in maternal health outcomes, in that most maternal deaths occur in rural communities [5]. Disparities are often the result of the unequal distribution, physical inaccessibility and financial inaccessibility of adequate maternal healthcare services and infrastructure in rural Nigeria [6–8]. Physical inaccessibility refers to distance, transportation, infrastructural, topographical, and resource availability-related barriers to receiving facility-based care, while financial inaccessibility can include high costs of transportation to facilities, high costs of medical supplies and services, and high costs of emergency care. The provision of maternal health care is the responsibility of three tiers in the hierarchical system. The first point of contact and main source of formal maternal healthcare services, which refer to evidence-based, maternal health services provided by accredited health professionals, is a primary healthcare center (PHC) [9]. Rural populations are significantly underserved in Nigeria, which highlights the inequity in their ability to access and use adequate PHC services, and ultimately the higher likelihood of maternal deaths in rural Nigeria [8, 10]. Accordingly, rural women in Nigeria use modern contraceptives less and have more abortions, and receive far less formal antenatal, childbirth and postnatal maternal care than urban women, putting them at higher risks for maternal mortality [3, 11]. The continuation of the current trends in healthcare utilization amongst rural populations will impede Nigeria from meeting SDG 3.1 by 2030.

According to Moore and the World Health Organization [12], respect for elders, approval by elders, and adherence to elders’ advice is traditionally believed to be important in rural communities. In many rural African communities, chiefs and other community elders act as the main opinion leaders and primary decision makers, exerting the most influence on the daily life of community members [12, 13]. In the context of maternal health, such stature at the community, household and even individual level enables elders to hold traditionally-sanctioned influence over care-seeking women and their decisions from family planning to puerperium [13–20]. In parts of Nigeria, women’s decisions on maternity care are largely within the traditional purview of leaders in the household and/or in the local community [21, 22]. Community perceptions about health programs and health services affect utilization of health facilities [23]. The perspectives and beliefs of elders can therefore have a critical influence on whether women seek and utilize evidence based maternal care. Their influence on maternal health indicates that Nigeria must incorporate influential community elders in maternal health strategies to help push towards the SDG 3.1 target [24].

Currently, the lack of evidence and poor understanding of the perceptions of influential elders on maternal health contributes to the poor maternal healthcare development, promotion, access and uptake in many rural communities. Consequentially, this has hindered the impact and success of national, regional and local maternal healthcare programs and services, and thereby the improvement of maternal health outcomes throughout Nigeria. This study explored community elders’ perceptions on the poor use of formal maternal care by women and causes of maternal death in rural communities in Edo State, Nigeria. It also aimed to identify potential solutions that can increase utilization of evidence-based maternal care and reduce maternal mortality. Literature on community interventions indicates that mobilizing community members to take charge of needs and tailoring programs to address identified community needs can increase their local acceptability and effectiveness [25, 26]. The study will help us understand the local challenges, needs, and priorities, as well as the support that communities can provide for women to better access and utilize facility-based care. In turn, this can help inform new or existing interventions and increase their acceptability and effect in targeted Nigerian communities. Ultimately, findings will help improve the utilization rates of evidence-based maternal care and reduce maternal mortality in study communities, and thereby Nigeria as well.

## Methods

### Study design

The qualitative data reported in this study were extracted from within a larger, original project being carried out in Edo State Nigeria by The Women’s Health Action Research Center and the University of Ottawa with the aid of a grant from the Innovating for Maternal and Child Health in Africa initiative- a partnership of Global Affairs Canada (GAC), the Canadian Institutes of Health Research (CIHR) and Canada’s International Development Research Center (IDRC). The goal of the project is to reduce maternal mortality in Nigeria by strengthening the availability, accessibility, and use of maternal primary health care services by vulnerable women. The project is designed as a community-based, multi-site, and multi-disciplinary cluster randomized trial that uses a mixed methods approach. It was designed to maximize community participation and ownership in the design and implementation of community-based interventions across the country. This paper focuses on and reports findings on elders’ perceptions on maternal healthcare utilization and maternal death, which was a component from the qualitative segment of the project. A qualitative approach with a phased analytic plan that elicits themes was employed. This study was reported based on the Consolidated criteria for reporting qualitative research (COREQ) (see attachment**).**

### Research setting

Nigeria has a population of over 190 million people, making it the seventh most populous country in the world [27]. With one of the fastest population growth rates in the world, Nigeria has a total fertility rate of 5.42 (live births per woman). Nigeria’s population is projected to rise to 411 million by 2050, which would make it the third most populous country [27]. About 50% of Nigeria’s current population is rural [28]. Edo State, which is in the South-South geo-political zone, is one of Nigeria’s 36 federating States. It has approximately 4 million people residing in 18 Local Government Areas (LGAs) [29]. Two of the predominantly rural LGAs in Edo State have been selected for this study: Esan South East (ESE) and Etsako East (EE). Located in the riverine and rural parts of the state, the two LGAs combined for a projected population of 399,917 in 2015, with ESE accounting for a projected 212,055 and ETE accounting for a projected 187,862 [30]. In addition to their rurality, these LGAs were selected following the preliminary baseline assessments of the larger project due to relatively high maternal mortality rates and low PHC utilization rates among Edo State LGAs.

### Participants and recruitment

At the baseline stage of the larger project, a geographic mapping of different communities was conducted during a preliminary and scoping survey in ESE and ETE. As the first points of contact for maternal care, PHCs in ESE and ETE were identified. Nine study communities determined to have traditional age-based hierarchies across the two LGAs were selected for community conversations, with 4 having a local PHC and 5 not having a local PHC. Positive social changes in communities require the identification and incorporation of the community members who have a significant influence on local decision-making [31, 32]. For this study, community elders (≥50 years of age) who were locally recognized as influential opinion leaders were the targeted participants. Their position in the traditional hierarchy can help garner support for community initiatives, influence modernization of traditional beliefs and practices surrounding maternal health, and improve the acceptance, effectiveness and success of maternal health programs.

Study participants were recruited through purposive sampling using locally accepted methods of communication, which included meeting community chiefs or traditional rulers before commencing recruitment of community members. Accordingly, purposive sampling helped to ensure inclusion of elders who were considered local health influencers and motivators. First, IDRC-affiliated project leaders identified trusted indigenous guides in each community, who then introduced the project and the IDRC-affiliated local research team to the traditional ruler of their community. Afterwards, the local research team met with the traditional ruler of each community to explain research purposes, to obtain consent for the research, and to request a meeting with elders. Community rulers scheduled meetings with community elders for data collection and helped introduce researchers to the participants. Recruitment of elders was continued until data saturation was reached [33].

### Data collection

This study conducted community conversations (CCs) with community chiefs and other elders who have a substantial influence on local practices. A CC involves members of a community coming together and holding discussions about a concern, followed by the construction of resolutions to bring about social changes [34]. In accordance, this form of data collection has been found to be effective in some African communities in resolving difficult social problems and getting affected communities to control the process of change relating to those problems. CCs have helped raise awareness and address a range of issues, such as the following: HIV testing and prevention, female genital cutting, and child marriage [35], as well as mental health stigma among ethnic minorities [36] and health issues in rural Native American populations [37]. An assessment of CCs as a community engagement tool found that the method helped increase awareness among community members, provided a voice for members to share concerns, and facilitated discussions about significant topics [38]. CCs effectively created a participative environment, promoted relationship-building and collaboration among community members and between community members and external stakeholders in discussing potential solutions to identified problems, as well as planning future actions [38]. In rural communities, CCs are especially common and effective for transferring information, driving social interactions and change, and altering local beliefs [34, 37, 39].

For this study, the conversations were designed to enable elders to share and discuss their views and concerns about maternal mortality and use of facility-based maternal health care, as well as to proffer potential solutions. These conversations helped to identify local needs, priorities and the support that communities in the LGAs can provide and require for women seeking evidence-based services. Proposed solutions to the identified maternal health problems lay a foundation for intervention components that would be acceptable to specific rural communities. The CCs were conducted in Pidgin English and a few in local languages (Ishan and Etsako) by trained IDRC project-affiliated investigators, including FO and LN. During the baseline phase, prior to the formative phase of the project, a baseline study was conducted in 20 randomly selected communities in Etsako East and Esan South East LGAs (10 from each LGA). Nine study communities were selected from the 20 communities based on the presence and residence of influential elders, as well as the traditional rulers ruling these communities. A total of 9 CCs were conducted with 6 in ESE and 3 in ETE. The number of participants in each CC ranged from 12 to 21, small enough to allow all members to speak, but large enough to maximize conversations from elders with different opinions. The CCs were conducted outdoors by the means of a CC topic guide designed to gather perceptions about maternal health related topics. The guide was developed by a technical committee in charge of preparing research instruments for the larger project. The members of the committee were familiar with the cultures of the project communities and the pertinent questions for the conversations. All the research instruments and procedures, including the CC topic guide, were piloted in a suburb of Benin called Oluko with 12 men (≥50 years of age). Meetings had facilitators who guided the conversations with the topic guide, which was also designed to involve the participants in problem solving. The facilitators were IDRC project- affiliated field supervisors who held traditional positions, such as chieftaincy, or were conversant with the traditions of study communities. These facilitators were experienced qualitative researchers who spoke Standard English, Pidgin English, Ishan and Etsako. Facilitators received project specific training in qualitative data collection and in facilitating CCs before field work. FO and LN were senior IDRC project investigators who oversaw the recruitment and data collection stages in ESE and ETE.

At the start of meetings, traditional methods of meeting with the community leader were used, including the sharing of kolanuts and requests for traditional prayers for research success. The reasons for conducting the project were then explained, after which the elders were engaged to share existing problems in maternal care. They were encouraged to partake in creating solutions to identified problems and in community relevant and appropriate action plans to help improve maternal healthcare utilization. Discussions in the CCs lasted for approximately 90 min to give all the participants a chance to express their thoughts. The discussions ended when no further topics arose (point of saturation). After closure of the meetings, resolutions were itemized and read to the elders for respondent validation. The elders reviewed the resolutions and thereafter gave feedback on the itemized resolutions. Proceedings in the meetings were audio-recorded, transcribed and reviewed for clarity and accuracy of the transcription. Participants’ responses were either transcribed verbatim if they responded in English or translated if they responded in Pidgin English and one of the local languages. Literal translation (word-by-word) was used to preserve the participants’ responses and provide readers with an understanding of their mentality [40]. They were assigned codes to remove any identifying information that could jeopardize their anonymity and privacy.

### Data analysis

Prior to commencing analysis, audio-recorded conversations were transcribed with the assistance of translators. Data from transcripts were analyzed through inductive thematic analysis using NVivo 12 software. Braun & Clarke’s [41] guide for conducting a thematic analysis was followed as it enables a transparent and rigorous analysis that produces pertinent information required for the study’s research approach. The theoretical flexibility of thematic analysis enabled us to analyze different aspects of the research objectives, developing or extending understanding of elders’ perceptions. It also helped reflect the richness, the detail and the in-depth nature of the qualitative data collected in the study [41]. The primary and corresponding author independently read the transcripts repeatedly to get immersed into the raw data and make note of initial topics and ideas relevant to the research question. The transcripts were coded in an iterative manner, revisiting the transcripts and altering and modifying the codes as reflected by the data and the emerging patterns. Excessively detailed word-by-word or line-by-line coding reduces the ability to see patterns among and between pieces of data [42]. Lines of text were thereby coded broadly, often ranging from a sentence to several sentences, to ensure that the intentions in the participants’ views were not lost. Many references under each code also included some surrounding data to ensure the context of meaning was intact, acknowledging that some texts can be categorized into different codes. The primary author (AWF) and corresponding author (SY) then discussed their codes and resolved any differences in coding, after which a final consensus agreement was reached. Themes and subthemes were developed from the codes and the dataset after making sense of the patterns in the coded data relative to the research question [41]. The final themes were validated and were accepted as being representative of the data within the context of the research question. The final themes were named to tell the story of the categorized codes. Selected quotes in the reporting of findings are chosen to represent a typical response relative to the reflected theme. Given the inductive nature of the data analysis, saturation was achieved when no more codes or patterns emerged from the data.

### Trustworthiness

Trustworthiness of qualitative research is crucial for ensuring a rigorous study that produces findings capable of making an impact on policy or practice [43]. Multiple authors are involved in data collection and analysis. Following data collection from the CCs, FO and LN employed member-checking in order to receive validation and ensure credibility of the proffered solutions. Multiple coders (AWF and SY) were used to independently code the data and then to collaboratively refine their proposed codes and thematic patterns. Feedback was received from field investigators FO and LN, who have ample experience in reproductive health research in rural sub-Saharan Africa and are involved in the larger project as principle investigators. Clarifications, project issues, thematic misinterpretations, contradictions, factual errors, and reporting of study findings were raised and discussed. A colleague with qualitative research experience was also engaged by the primary author to serve as an external auditor and further ensure dependability.

To ensure confirmability, the decisions made in the research process starting from the research objectives to the interpretation of findings are thoroughly described, along with examples of data to support findings and conclusions [44]. Data is collected from male elders and female elders, the latter having had more direct experiences with maternity in their life course. Data is also collected from multiple locations in the two LGAs, thereby involving different elders in each community. This data triangulation helped enrich and deepen the understanding of study findings [43, 44].

### Ethics

Ethics approval for the larger project was granted by the National Health Research Ethics Committee of Nigeria (NHREC) – number NHREC/01/01/2007–18/04/2017. Ethics approval for this qualitative study was received from the University of Ottawa Research Ethics Board (REB) on 18/03/2019. Participants were voluntarily enrolled in the study on the basis of a free and informed consent. Participants were informed that information collected from the research project would be used to understand the current community needs, to improve the future usage of evidence-based maternal health services, and to improve maternal health outcomes in their community and Edo State. Participants were then informed that once they chose to participate, they could withdraw at any time and/or refuse to answer any questions, without suffering any negative consequences. Permission to audio-record the community conversations was sought and obtained before data collection. Processes for managing and storing the audio files from the CCs were put in place to further ensure confidentiality of study participants. All personal identifiers were removed from transcripts and in quoted texts below. However, participants were informed that information shared in CCs is exposed to other participants and may be a limit to their overall confidentiality due to the inability to completely control the actions of others. Written informed consent was obtained from all participants prior to their participation.

## Results

### Characteristics of study participants

A total of 151 men and 7 women aged 50–101 years of age participated in the community conversations. Most of them attained post-primary education, whereas a few had no education. The majority were farmers and artisans. Majority of the participants were Christians, and a few declared no religious affiliation.

### Reasons for underutilization of formal maternal care

#### Quality of care

The elders mentioned various reasons related to quality of care, perceived or actual, that contributed to the reduced uptake of facility-based care. A recurrently stated reason was understaffing in health facilities, and the corresponding inability of such facilities to meet the needs of their clientele. The lack of health professionals in PHCs and even some hospitals was a major deterrent. Several elders exclaimed that understaffing issues were the consequence of posted nurses and doctors skipping their work duties at the facility. The absence of nurses and doctors prevented community members from receiving skilled care from health professionals:

*“this is Nigeria, it is poorly equipped, even the so-called general hospital, I can’t say it’s a no go area, but we all know what happens there when you get there, it’s either the doctor is absent or the nurses are absent”* (CC 02, ETE, Male).

*“may God keep you all, the health center that they said is here there is no nurse where three nurses are supposed to be on duty - it is only one nurse you will see, in a week you will not see them - if someone sustains any injury and is rushed there you will not see nurses unless you go to the next community which is Ewohimi or Ubiaja for treatment”* (CC 08, ESE, Male).

Elders expressed their frustrations with the perceived unprofessionalism of health professionals, including those who were absent from workplace duties. They criticized them for not seeming to take their jobs seriously, and instead carrying out personal tasks, such as shopping, during work hours:

*“the habit of absenteeism is very common among them let’s say you ask a nurse to wait for you she will go to the market until the later hours before she comes back or until the next day. For example, there was a patient brought to the health center, there was no nurse to give treatment. The next thing was to take the person to the nearby chemist [collective laughter]” (*CC07, ESE, Male).

Some health professionals were said to display patient favoritism when deciding which patient to treat first. There were also accusations of financial status discrimination in which patients with higher wealth status and influence received more prompt treatment and attention than patients with lower wealth status. Nurses were particularly accused of not following protocol and fulfilling duties, including referrals of patients to their own homes for care and abandonment of their facility duties during work hours:

*“It is not because of the charges, I have never seen anyone who comes back after good care and complains that the money is too much and tells other women not to go. The reasons are the nurses are not always on duty for their primary assignment, and even if they are there on duty they will take you to their home for treatment or they will refer you to a place where by the time you get there, it is the same person who referred you that you will meet there”* (CC 07, ESE, Male).

Some patients who were rushed to a health center due to an accident were said to have arrived to a facility with no nurse attendants. At the nurses’ homes, even when drugs were not present or proper for the required treatment, referred patients were sometimes asked to pay regardless of treatment effectiveness. Accordingly, nurses were also accused of partaking in drug trafficking by taking facility drugs to their home and selling them off to certain people. Many PHCs were further perceived to provide poor and inadequate care because of building erosion, poor sanitary conditions, bat infestations, lack of lighting, lack of boreholes and water, and lack of toilets. Poor health facility conditions were believed to contribute to issues with provider retention and resultant staff shortages. Bat infestations were a specific reason some nurses and midwives refused their postings in certain PHCs, according to a male elder:

*“I remember when they posted a nurse to this health center, she refused to go be posted, her reason is because there is a bat in the facility. The problem the bat brings is that it emits worms from its feces, it would be falling into their health center, so the nurse refused to go there when they transferred the other woman. She said her health is more important than any other thing, she said she does not know what the worms can do, and also the smell of the feces”* (CC 05, ESE, Male).

In contrast to health professionals, the constant availability of traditional birth attendants (TBAs) made them local favorites amongst service users, including those with the financial means to use a health facility. TBAs were non-health professional attendants who were often older female community members with experience in providing native (traditional) care to mothers throughout the continuum of maternity. TBAs were able to provide native maternal care to women in the service users’ homes or in traditional maternity centers. This type of care could range from providing advice and social support to pregnant women or new mothers, to assisting homebirths, to performing cultural rituals during any maternal period.

In addition to personnel shortages, some PHCs and hospitals were also thought to provide inadequate and improper maternal care due to shortages in medical equipment and drug supplies. Community members who wanted to receive facility-based care were sometimes forced to go to another PHC in order to buy drugs. There was skepticism as to whether this was due to drug supplies being diverted by health attendants for their own use or if the facilities were generally undersupplied. Long wait times in health facilities, which were caused by health professional shortages and overwhelming demand, encouraged some community members to seek and opt for native maternal care instead. Long waiting times in health facilities were a source of dissatisfaction and another key factor in the low uptake of facility-based care. Conversely, native maternal care was associated with prompt, appropriate and attentive care.

Provider incompetence in providing care was voiced to be another knock on the quality of care in health facilities. Many elders were of the opinion that nurses and doctors in health facilities were underqualified and thereby not fully capable of providing high quality maternal care to women. Additionally, nurses were perceived to lack knowledge of how to use new medical equipment:

*“we have a facility here, but we don’t have good nurses and doctors who take care of our pregnant women. Though they are trying, we need to have more qualified people. Sometimes when you go there and they want to give an intravenous injection, they struggle to see the vein”* (CC05, ESE, Male).

*“all these things I mentioned, even the so called nurses were seeing them for the first time, so of what use is this plate to you, when you don’t know how to use it (some individuals laugh). For example, the suction machine, the nurses there, I don’t think they have ever used that equipment since it was brought there, there was another machine there, that is supposed to be use for, when checking sugar level, the nurses there I don’t think they know how to use it”*(CC 02, ETE, Male).

Interpersonal relationships between patients and health professionals were key talking points in the CCs. Health facility staff, namely nurses, were alleged to be uncooperative and rude to their patients. After questioning the employment of a poorly mannered nurse, a male elder stated:

*“by the time she came, she started talking so mannerlessly that I don’t know how she got her job so that is the more reason people don’t patronize them as such. I witnessed a case where the nurse was telling the woman was I there when your husband impregnated you, did you not enjoy the sex, so if you can’t pay the money I will not render you any services. This is what is currently happening in the state and everywhere, please you people should caution the health workers here”* (CC07, ESE, Male).

In contrast, the relatively positive relationships with TBAs or other informal attendants encouraged community members to seek out of facility care, irrespective of cost differentials. Traditional care-takers were deemed to be more hospitable, caring, and supportive, qualities that attracted some community members towards native care and pushed clients away from facility-based care.

#### Accessibility

In several communities without a local PHC, access to and utilization of facility-based care was significantly hindered, with the nearest PHCs being in other communities. The distance to a PHC was thereby a major physical deterrent to facility-based care. This was especially the case for those without a local PHC, who had to travel to neighboring villages to access formal maternal care from a PHC. The absence of a local PHC was said to force some women to opt for native maternal care from local TBAs, who were often nearby and readily available. Long distances to a PHC, whether local or in another community, was believed to be the most significant barrier to women who experience emergencies, such as from premature labor and births, and need to reach a facility as quickly as possible. One male elder explained:

*“Just like my brother said just now, if an obstetric emergency happens, it is not easy to rush the woman to the PHC for emergency maternal care. The situation here is that our source of maternal care is very far from here and we have no road to access the facility”* (CC 03, ESE, Male).

Difficulties in finding the means and modes of transportation to a PHC and receiving professional assistance were also discussed as a hindrance. Women who go into labor late at night or who require immediate emergency care were said to be most affected by transportation constraints. Others identified poor road infrastructure as a key barrier to accessing health facilities. They stressed that even with a physically close PHC, existing poor road conditions would hinder their community from physically accessing the facility:

*“We don’t have a clinic here, and for the available one in another community, we don’t have the road infrastructure to even access it, this is causing us suffering” (*CC 09, ETE, Female).

The unaffordability of care was perceived to be another obstacle for those who wanted to receive skilled maternal care. The costs started at home where they would need to pay for transportation, such as a motorbike, to get from their residence to the health facility. At the facility, high costs of health services and equipment were said to restrain some community members from receiving maternal health services:

*“Yes the charges are too high because here when a woman gives birth to a male child, they charge 10,000, and when they give birth to a female child, it is 8,000 so it is high. That is why we decided not to go again, we don’t have that amount to be spending, and since you people want to come to our aid we are so happy”* (CC 08, ESE, Male).

Community members that sought and intended to use formal care were predominantly inhibited by aforementioned constraints in proximity, transportation and affordability. However, many women preferred and opted for native treatment because it was perceived to be less risky than relying on facility-based care. Additionally, native treatment with the assistance of traditional attendants was cheaper, and more convenient and pragmatic.

#### Lack of knowledge

Fears of medical operations on a woman’s health and well-being pushed some women to opt for native maternal care from traditional attendants, thinking that the avoidance of health facilities would help prevent complications and operations. Women were said to only register and visit a PHC when they felt weak, seeing the facility as a mere source of treatment for when problems arise. Women were also believed to lack knowledge and understanding of family planning, including of where to receive family planning care before pregnancy or after childbirth. Considering the number of children women conceived, some were said to set the upper limit at whenever they felt weak or too tired to give birth to additional children. Others were said to follow native family planning in which they kept trying to conceive with the belief that God would cap the number of children they are meant to bare. Elders contested that women who opted for such native care have limited knowledge, despite thinking they know a lot.

### Perceived reasons for maternal death

#### Medical causes

In the CCs, malaria was perceived to be one of the causes of maternal death during pregnancy. Pregnant women who were infected with malaria were alleged to be more difficult to treat than non-pregnant women who were infected with malaria. They explained that some drugs taken in the past for malaria by women in a non-pregnant state became dysfunctional when taken during pregnancy. There were also elders who believed that maternal mortality is caused by excess displaced blood in the pregnant woman’s body.

#### Facility service deficiencies

In reference to women who undergo labor and require immediate medical care, PHCs that were not operational overnight were believed to contribute to their potential deaths. Women who needed to deliver had to opt for self-care at home or care near home from a non-professional attendant, namely a TBA. Others believed that inadequate drug supplies contributed to maternal sickness and possible death. The unavailability of drugs especially impacted the timely care of emergency obstetric situations.

#### Native maternal care

During pregnancy and childbirth, many women preferred and opted for native treatment with native herbs over medical intervention and professional assistance in a health facility. The death of some women who opted for native treatment led to beliefs that utilization of native care over medical care was the major cause of maternal death. Native maternal care was associated with trial and error treatments, which made it undependable.

#### Poor awareness and negligence

For some elders, maternal death was ascribed to poor awareness of the significance of professional care during maternity and the seriousness of the maternal health risks. Women’s negligent disregard of health instructions was also associated with maternal death. Many purportedly opted to stay at home instead of going to a PHC for the recommended checkups, unless an abnormality occurred. Women were said to snub advice about family planning and physical work during the early trimesters, thereby increasing the burden on their bodies. Some women also used malaria nets for farming purposes instead of their original purpose in protecting against malaria infection and the associated ramifications for the mother and fetus. A man spoke about women who prefer native herbs:

*“Regarding the issue with the causes of death of pregnant women, it is because they do not follow instructions. Most of these women when they are pregnant, they don’t like to use the hospital, because even though there are specialists there that are properly trained to take care of them, instead of going to the health center, they prefer to take native herbs”* (CC 05, ESE, Male).

### Proposed solutions

#### Improve access to adequate facility services

Majority of the participants in communities with no local PHC recurrently proposed the need for a local PHC or a closer hospital. A local health center was perceived to mean faster access to skilled labor and delivery assistance, especially during emergency situations. It was also favorably associated with shorter distances and convenience, lower transportation fees, easier modes of transportation, and the capacity to serve surrounding communities. Ultimately, a local health center was believed to increase access and use of health professionals and reduce the number of maternal deaths:

*“if a health center can be built here, it will facilitate the whole issue for our women to meet with the health worker. This can also help because the one we have is situated at Eguare, if we can have a centralized one here it would help us to help our women and it will also make it possible for other nearby communities to make use of it because the one we currently have is far”* (CC 01, ESE, Male).

*“if a pregnant woman is in labor, if the woman is rushed to Ubiaja, the next village, before she gets there she may have lost strength and died. Also, to be rushed to the nearby health center just to go and deliver there is a whole other issue, please we need help in this our community”* (CC 08, ESE, Male).

In communities with easy access to a PHC or hospital, some elders stressed that facility conditions needed significant improvements in order to encourage facility uptake. The presence of a health facility alone was said to be insufficient by many participants who suggested increased availability of lighting, water supply, good equipment, and a variety of drugs for treatment in health facilities. To improve accessibility, several participants also recommended operational, round the clock PHCs or hospitals that would be open at all times. It was proposed that several health professionals be designated alternating shifts to operate a 24-h functional health facility. For understaffed PHCs, scheduled provider visits were suggested where certain health professionals would be stationed at the local PHC on specific pre-determined dates:

*“if you know that a doctor is coming to the health center by Wednesday at least to attend to the pregnant women and children, you understand what am saying, then every woman and pregnant child will now know that doctor is coming today and they will acknowledge that they should not go to the farm on that day. They should be ready to go and see that doctor and present my case instead of going to the general hospital in Agenebode where we don’t know if the doctor has travelled”* (CC 02, ETE, Male).

In areas where physical access to a destination was hindered by topographical barriers, some participants implored the desperate need for road repairs. One participant expressed how building a health center alone would not make it accessible:

*“You can see how easy it was when you were entering here. There is no road, people can hardly access it. Even if you build a health center here, is it not the road people will still have to pass? So if you help us repair the road, we will really appreciate it”* (CC03, ESE, Male).

Improvements in the technical and interpersonal quality of health professionals were recurrent suggestions. Participants principally asked for their health facilities to be staffed with qualified health facility staff that can provide adequate maternal care. Across communities, the poor quality of care from health professionals was believed to be related to poor training. In view of that, participants recommended training regimens to improve the quality of health facility staff, including training to improve referral capacities. Speaking about the nurses who struggled to use the suction machine, a participant spoke about training:

*“so what am saying in essence is that these nurses themselves who are supposed to be the ones helping us, they need help because to be trained, they need to be up to date with the recent equipments you have in the world today, they need to update themselves”* (CC 02, ETE, Male).

Participants also suggested inspections and audits of health professionals to assess whether they are doing their jobs and behaving properly. Consequential punishments were correspondingly suggested so that other health facility staff can learn their lesson:

*“set people up to monitor them, if they are not on duty, punish them. I think other health facility staff will learn their lesson. This happened among the teachers, but now other teachers have learnt their lesson, so if it can also happen in the health sector, they will also get their lesson”* (CC 07, ESE, Male).

Another recommendation was to help locals acquaint with the health facility staff so that they can know the people that are working in the facilities and how they function. This acquaintance was especially believed to help locals receive primary treatment from attendants who allegedly attended patients by order of favoritism. With frustration over attendants’ prioritization of money before treatment, it was suggested that the patient’s health, well-being and care should be attended to first before discussing money.

#### Health promotion and education

As a resolution for poor awareness of maternal health, participants across the two LGAs implored that community members, primarily women, be educated and enlightened. Some participants believed they needed lessons on the differences between facility-based care and native care, as well as the significance of professional attendance. Enlightening women of the necessity of professional care was believed to improve the uptake of health facility services and improve maternal outcomes:

*“the issue of family planning is for women and most women here have not heard of it, so what I think can be done is to create awareness for them to have better knowledge of it”* (CC 07, ESE, Male).

*“I think they should be enlightened, they should know that during pregnancy they are to register with the hospital, not to seat at home to enable the nurses to monitor the women and the baby till delivery day. If we continue like this the rate of death in mother and child will be reduced”* (CC 01, ESE, Male).

Regarding the educators, some participants proposed appointing community contact persons who would be lectured by health professionals, and thereafter relay the learned information to their community. A few proposed that health professionals come directly to their community, where they would educate women on maternal health and how to reduce the likelihood of death. Some participants alternatively proposed educating men first so that they can spread knowledge and awareness to females at home, including on when to refer their wives or daughters to the health center. Community education of the proper use of health measures designed to protect women and their children from malaria was also discussed. Accordingly, education on the proper usage of malaria nets and fines for the continued misuse of malaria nets were recommended.

#### Community support

Several proposals were made involving community support as solutions for improving the ability to receive services from health facilities. Community insurance plans involving gradual payments by community members were mostly discussed and recommended, with an insurance provider, such as a trusted intermediary, managing the contributions. Others discussed and endorsed co-sharing in which community members would loan money to others, particularly to those in urgent need of evidence-based maternal care. When needed, the insurance provider or money loaners would partially or fully pay for maternal services and improve the immediate financial accessibility of facility-based care. A few male elders also proposed that men be active and involved in health-seeking to make sure women have professional care at a facility. Elders in communities without a local PHC discussed the need of land for the construction of a local PHC. Some participants proposed to find and decide on land they would be willing to give up for the construction of a local PHC. In one community, participants proposed to provide a vacant building in their community for free, so it can be restructured into a PHC. Finally, they proposed that community members would be willing to provide hands-on assistance to builders of local PHCs:

*“We have land here in this community that we can give to you to build the facility and we the community members will also join hands with you to build the structure in unity, because when two rats join tails together, it will be as thick as that of the rabbit (parable). We will join hands together to make sure that you are able to do the project, that is the joy of our community.”* (CC 04, ESE, Male).

In communities with local PHCs, some participants proposed community members provide assistance in maintaining the physical conditions of the facility. For example, in the PHC where bats were creating issues in provider retention, community members were willing to spray chemicals to rid of the bats.

#### God’s assistance

Some elders believed that God would help women throughout pregnancy and childbirth. They also expressed that God would provide power, guidance, and assistance to health professionals and researchers to help community members. A few elders claimed that God was a solution for preventing maternal deaths. One female participant alluded to her belief that health and healing were up to God regardless of the presence of a health facility:

*“even if someone resides in a cave, our God will still raise a helper who will locate that person inside the cave… it’s only God that is helping women in this community. God should create a way for you people to help them in this community”* (CC 09, ETE, Female).

## Discussion

The study has explored and provided insight into community elders’ perceptions about reasons for the underutilization of facility-based maternal care and reasons for maternal deaths in rural Edo State. The results demonstrate that elders perceive a wide range of factors that contribute to maternal deaths and low utilization rates of facility-based maternal care.

The perceived reasons for maternal deaths were related to medical factors, supply shortages, inoperative facility services, uptake of native care over formal care, and poor awareness and negligence of maternal requirements and risks. Previous studies on perceptions of reasons for maternal death also identified medical and nonmedical causes believed to lead to the outcome [45–48]. Study results indicate that elders held narrow perspectives of potential medical reasons for maternal mortality; there were discussions on displaced maternal blood and malaria, but no mentions of other major medical factors in Nigeria, including other infectious or transmissible diseases, sepsis, obstructed labor, and unsafe abortions [3, 11]. Studies in rural and urban Nigeria reported that policymakers, elders and other community members perceived malaria or fever as the most common medical ailments leading to a maternal death [45, 49]. Policymakers and male partners in sub-Saharan Africa believed excessive bleeding was the most common direct cause of women’s maternal death [49, 50], which is similar to narratives about bleeding in this study. Non-medical reasons identified by elders in this study include unavailability of facility services, and poor awareness and negligence. These reasons are related to non-medical determinants of maternal deaths identified in the literature, including social, economic, and cultural factors [45, 49, 51–54], as well as political factors, healthcare system coordination, health services provision, community contexts, and demographic characteristics [49, 55]. Delays in reaching health facilities, delays in receiving care, and poorly skilled health attendants were also held responsible for high maternal mortality rates [50, 54, 56]. A cross-sectional study in Nigeria reported that men blame women’s failure to use FP, emergency, antenatal, and delivery care services for their deaths [54]. This resonates with narratives from many male elders who mostly pinned maternal deaths on women due to their lack of knowledge and negligence. Additionally, women’s uptake of native maternal care over facility-based care was cited as a reason for maternal mortality in Edo State. The uptake of native maternal care has been strongly associated with higher rates of maternal mortality and other poor maternal outcomes [49, 50, 57]. Non-professional attendants, namely TBAs, lack the basic knowledge and skills required for the adequate provision of evidence-based maternal care [58, 59]. Additionally, TBAs cannot manage obstetric complications, increasing the risk for poor maternal outcomes. On the other hand, the use of evidence-based and professionally assisted care reduces the likelihood of poor maternal outcomes [57]. Rural women in Northern Africa believed lack of maternal health awareness was a cause of maternal mortality [60], which corroborates the accounts given by elders in this study.

Elders voiced that women did not utilize facility-based healthcare services for a variety of reasons. Firstly, many were hindered by the unavailability of a local health center, health professionals, adequate facility infrastructure and conditions, transportation to facilities, and drug and equipment supplies. These findings are consistent with previous findings across rural Nigeria [61–63] and other rural African settings [64–68], from research that identified availability as a key deterrent to use of facility-based services. In contrast to PHCs and hospitals, traditional maternity centers and traditional attendants, as well as self-care in one’s own home, were more readily available options and thereby more accessible than facility-based care. Poor technical abilities, poor communication with impersonable health facility staff, and unprofessional acts from health facility staff contributed to the perceived low quality of care in health facilities, which is a deterrent to facility-based care, as seen in other rural settings [62, 63, 65, 69–72]. Similar to study findings, a study in rural Tanzania found that corruption among health facility staff was rampant, with attendants asking for bribes in order to provide optimal care, and threatening to provide suboptimal care for those who did not offer any bribes [68]. Poor road infrastructure, long distances to facilities, and high costs of transportation and health services are major deterrents to the use of maternal healthcare services [45, 48, 61, 63, 67, 69–74]. This is corroborated by findings in this study where geographic and financial constraints to health facilities were believed to lead to the non-utilization of health facilities. Although it was not identified as a factor in this study, the lack of payment options and the requirement of payments before treatment have been found to drive service users away from facilities and towards TBAs [75, 76]. TBAs desirably enabled non-monetary methods of payment and provided flexible time frames for repayment, unlike health facilities. The elders also perceived poor community knowledge and understanding of basic maternal health requirements to influence the choice to opt for native maternal care. In the literature, poor education of women, partners and household leaders about reproductive health and care-seeking was identified as a major deterrent to the uptake of facility care services [66, 69, 77–88]. Moreover, systematic reviews exploring barriers to the access and use of facility-based obstetric care in sub-Saharan Africa found that lack of information on healthcare services and providers among community members contributed to poor knowledge and awareness, and thereby the reduced uptake of formal maternal care [89, 90]. Other findings from the reviews however were not reported to be barriers to facility-based care by elders in ESE and ETE: young age; unmarried or single; previous uncomplicated pregnancies and births; cultural beliefs and practices; pre-occupation with household and sustenance duties; social stigma; lack of women’s autonomy; poor male involvement [89, 90]. Contrary to perceived reasons for underuse and death in the study, in some rural communities, utilization rates remained low despite high awareness and knowledge of maternal healthcare in the community [91, 92]. The implication being that in some rural communities, it is often multiple factors that shape health-seeking behaviors and utilization patterns. The priorities or ranking of the significance of deterrent factors can vary between community members, suggesting that resolutions and strategies must address multiple barriers to facility-based care.

To improve formal healthcare utilization and reduce maternal mortality, elders recommended several changes to, and solutions in, facility-based care. The first set of recommendations were to improve healthcare provision by building local PHCs, improving infrastructure in and leading to the facility, replenishing supplies of drugs and equipment, competence training for health providers, scheduled provider visits, extended facility hours, provider audits and corresponding consequences, and alleviation of financial costs. These suggestions generally reinforce strategies recently identified in prior research for improving primary health care services provision and use in rural Nigeria [93, 94]. Practical assistance from community members, the provision of community land and general community support were suggested. Evidence on community engagement and involvement has identified the key role communities can play in improving the quality of care and the use of skilled care, as well as reducing maternal mortality across rural Africa [93–96]. Community-based insurance and co-sharing were also recommended by the elders as a part of community support in order to help a member finance immediate maternal care needs. National and community-based insurance schemes have been widely proposed and successfully implemented across the developing world [97]. Moreover, the schemes have helped reduce financial constraints of maternal health services and helped reduce maternal mortality. Health promotion and education were recommended to remove misconceptions and improve community awareness, knowledge and understanding of available services, risks and danger signs, and the significance of seeking professional attendance. In addition to external educators, reliable contact persons within the community were identified to relay promotive messages, which are intermediary stakeholders that have been suggested in previous research [98].

As stakeholders with potential to influence women’s health-seeking behaviors, elders’ perceptions can play a vital role in facilitating uptake of facility-based care throughout the continuum of maternity, from family planning and antenatal care to postnatal care. Perceptions of other potential stakeholders who may influence or be influenced by maternal health actions, such as men, TBAs, healthcare providers, and women, can vary in each context based on existing challenges and needs, available resources, individual socio-cultural status, and values [77, 99]. For instance, a major factor of poor care quality by elders in this study can be directly experienced by service users, relatives, and care providers. Across sub-Saharan Africa, poor quality of care due to drug and equipment shortages, understaffing, or poor infrastructure was perceived to a major deterrent by women, husbands and male partners, and healthcare professionals alike [52, 68, 75, 100, 101]. Lack of local health facilities is another factor that can be experienced and identified as a major barrier by multiple stakeholders in a rural community in sub-Saharan Africa [75, 90, 101]. From the unique perspective of health professionals, issues from delays and overcrowding are often worsened by the lack of an appointment system and the random arrivals of women for maternal care in their health facilities [101]. On the other hand, health professionals are unlikely to identify their own behaviors and attitudes as reasons for the reduced uptake of their services. From the perspective of community members, including women, elders, and TBAs, negative facility attendant attitudes and behavior, as well as hostile facility environments, are viewed as strong deterrents to high-quality care and uptake of facility-based services [75, 101, 102]. Accessibility related issues that arise from far distances and inconvenience, costs of services and transportation, and unavailability of suitable transportation options to health facilities are also often identified as barriers by service users, relatives, and the TBAs who offer the more convenient and prompt alternative care [71, 75, 101, 102]. Although this study was conducted in communities that possessed traditional-age based hierarchies, the predominantly male elder participants did not perceive elders or male partners and relatives as sociocultural deterrents to women’s use of facility-based maternal services. Infact, discussions about individual and community level factors in the underuse of facility-based services and reasons for death were mostly focused on the women. From the perspectives of women, lack of decision making power and influence from relatives, husbands, elders, and other community members have been vastly identified as barriers to accessing skilled care [71, 77, 102–107].

### Strengths and limitations

This study included influential rural community members whose perceptions on maternal healthcare utilization and maternal death help to highlight community challenges and needs for adequate maternal care. The rich descriptions of their perceptions help to fill a gap in the research evidence. Another strength was the incorporation of rural community elders’ views, beliefs, and suggestions, which is significant for the development of locally appropriate and acceptable programs aiming to improve healthcare utilization and reduce maternal mortality. Though the primary author was not involved in data collection, the 2nd and 3rd authors were co-investigators of the larger research project and along with the corresponding author are well acquainted with the field of maternal health in rural Nigeria. Findings from this study should be interpreted in light of several limitations. First, community chiefs (leaders) were actively involved in the sampling of community elders who were believed to be influential opinion leaders. This could have introduced selection bias based on their personal preferences or interests, which could limit the dependability and authenticity of the data gathered from the CCs. Second, the study was not designed to assess differences across sociodemographic characteristics and thereby could not identify variations in perceptions by group characteristics. Third, even though the project aimed to capture a variety of perspectives from various elders, the results from 9 study communities in Edo State cannot be said to be transferable to all rural Nigerian settings nor to rural settings abroad. Every community will have different contexts, different existing resources and realities, and varying priorities when it comes to needs for improving healthcare utilization. Fourth, there were disproportionately more male than female participants in this study, as the larger project primarily targeted elderly men and thereby did not gather equal proportions of male and female elders. Influential elders identified by community chiefs and gatekeepers were also predominantly men, indicating that there are more male than female opinion leaders with influence in rural communities in Edo State. Therefore, represented perceptions may have been altered if more women were represented in the study, as they have more direct experiences with maternity. Fifth, some of the CCs were conducted in local languages (Ishan and Etsako) and later transcribed into English for analysis, which may have resulted in the loss of subtleties in language and nuances in meaning during the process. Future research that conducts analysis in local languages may identify different meanings in responses. Lastly, there was potential for recall bias when participants spoke of past experiences with maternal healthcare.

## Conclusions

Understanding the perceptions and beliefs of elders for maternal health services utilization is important in identifying ways to improve the provision of care and use of care, along with combating high maternal mortality rates. The findings of this formative study will help us to refine existing interventions and to design new additional interventions. This study also contributes to the currently very limited body of evidence on elders’ perceptions about the underutilization of facility-based maternal care and maternal death in the literature. It augments this limited literature by providing rich description of elders’ perceived reasons for facility-based maternal care uptake and maternal deaths. Congruently, this study confirms care quality, accessibility, and knowledge related deterrents to evidence-based care, as identified by various stakeholders throughout the developing world. The numerous reasons that were believed to have contributed to poor utilization of maternal health facilities and maternal deaths illuminated the various challenges communities can face in the fight to improve maternal health outcomes. The use of CCs to enable elders to form resolutions for community-wide challenges is a unique form of data gathering that has helped to elicit potentially helpful and locally acceptable solutions. Suggestions of community support in financing use of facility-based services and building health facilities are indicative of the willingness some underserved communities have in order to increase uptake of facility-based services. Additionally, suggestions to improve access to facility-based care and to provide health promotion and educational seminars highlight the multifaceted requirements of programs and strategies aiming to increase use of formal care and combat maternal mortality across rural Nigeria.

### Policy recommendations

With each community having its own needs and challenges, a series of interventions need to be tailored for each target community to have meaningful and lasting change in formal care use and maternal mortality rates. The series of interventions should not only be tailor designed at the individual and household level, but also at the community level to account for community contexts.

To increase the access and uptake of health facilities, political commitment, adequate budgetary allocation and funding into the optimal provision of facility-based maternal care is significantly required. This could include funding the construction of local PHCs, the refurbishment and upgrade of existing PHC infrastructures, and replenished supplies of drugs and equipment in PHCs with shortages. Issues with regular staff absenteeism, high turnover, and unaccountability highlight the poor management of health facility staff and the lack of transparency in care provision. Therefore, strategies that target attendant recruitment, retention, and accountability and adherence to workplace duties are direly required. Moreover, extensive training on capacity building, adherence to protocol, and necessary attitude and behavioral changes of health professionals is highly recommended to improve the quality of care. To facilitate the establishment of positive, respectful and hospitable environments in health facilities, health professionals need to be shown and offered adequate support by the health system, in conjunction with training on improved interpersonal communication with service users and their relatives. Periodic audits of PHCs and health facility staff can help to ensure proper and ethical provision of high quality care from health facility staff.

New or existing interventions should aim to remove or mitigate physical and economic barriers to access. This could entail building local PHCs, mobile maternal care services, or maternity waiting homes to help reduce distance barriers. Construction on road infrastructure could improve road conditions leading to PHCs and hospitals. Organizing transportation support schemes involving vehicles, taxi services, and/or motorcycle ambulances devoted for maternity care could help reduce transportation barriers. Developing national or community-based finance schemes could help reduce financial constraints, especially in communities that are willing to take part in insurance and loan schemes. An alternative recommendation is to provide free maternal health services or subsidized services based on income to ensure affordability of accessing and receiving evidence-based maternal care. Home visits and care could be provided for those who face significant obstacles in physically attending a health facility. Alternatively, scheduled provider visits to PHCs or hospitals could help to mitigate staff shortages and encourage user uptake of services on scheduled dates. Health promotion programs that educate rural community members about maternal health are recommended to improve knowledge and awareness of maternal health risks, birth preparedness, danger signs, and significance of evidence-based care for positive maternal and neonatal outcomes. A common concern that was implied throughout the study communities is the significance of trusted intermediaries for encouraging community-wide participation. Therefore, for trustworthiness, comfort, and thereby acceptability of health promotion sessions, trusted community members should be involved in community education implementation.

As opinion leaders, elders’ views and beliefs can be a vital source of information to inform policy and interventions aiming to increase uptake of facility-based maternal care and reduce maternal mortality. Considering their influence on reproductive health decisions in the household and the community to varying extents, interventional efforts should acknowledge and not overlook such influencers, especially in communities with traditional age-based or gender-based hierarchies. For example, interventional efforts aiming to promote uptake of facility-based care in communities with traditional age-based hierarchies can include opinion leaders in formative assessments of study communities, as well as the design and implementation of interventions. By actively involving such stakeholders from the formative to the implementation stages of community-based interventions, chances of acceptability and buy-in of the interventional efforts will increase. This resonates with the elders’ accounts of the significance of trusted individuals in facilitating positive actions towards maternal healthcare uptake. Interventional efforts based merely on the exploration of constructs such as preferences and perceptions of service users may not be accepted or effective in communities where women’s health-seeking decisions are considerably influenced by others.

With the worldwide infiltration of technology in this technological age, it would be remiss not to recommend implementing or improving digitalized health in some rural communities. As a matter of fact, digital health, such as through mobile phones, has proven to be revolutionary in the past decade in improving uptake of evidence-based maternal care in sub-Saharan Africa [108–113]. With the highest rate of growth in mobile subscriptions in the last decade, the use of mobile phones in sub-Saharan African countries has practically become a part of daily living [114]. Though the rapidly growing use of mobile phones has recently transcended urban-rural divides in Nigeria and across the sub-Sahara [115–117], increasing mobile phone ownership and closing the digital divide should be an aim for interventional efforts in the more isolated, remote communities with no access to phones or mobile networks. Mobile applications could be used to provide health promotion and educational messages to service users and relatives if necessary in order to tackle misconceptions, improve understanding of the significance of skilled care, and ultimately promote positive reproductive health behaviors. The information could be relayed via a wide range of methods, such as voice messages and pictographs in local dialects for users who are illiterate. Mobile applications could also crucially provide educational messages and training interventions to healthcare providers in order to improve their technical and interpersonal competence. Distance and transportation related barriers could also be mitigated by installing mobile-enabled digital communication platforms between users and the health system, which could be vital for connecting women experiencing emergency obstetric complications to ambulances or mobile health providers.

### Future research

Future research on health professionals’ satisfaction, recruitment and retention should explore their experiences and perspectives on the technical provision of care and interpersonal relationships with patients. This would help to identify the challenges and needs and the type of support health professionals require from their employers in order to provide high quality maternal care. Future research should also triangulate findings from elders with other community members to gather perspectives from participants with a wide range of experiences, realities and social positions. As this study is not designed to explore differences across sociodemographic and economic characteristics, future research should assess the relationship between participant characteristics and community perceptions. This would help to identify the sociodemographic and economic factors that may contribute to the uptake of formal maternal health services.

## Data Availability

The datasets generated and/or analyzed during the current study are not publicly available due analysis being underway for subsequent publications. They are available from the corresponding author on reasonable request.

## Abbreviations

CC: Community conversations
COREQ: Consolidated criteria for reporting qualitative research
ESE: Esan South East
ETE: Etsako East
IDRC: International Development Research Center
LGA: Local Government Area
MDGs: Millennium development goals
PHC: Primary healthcare center
SDGs: Sustainable development goals
TBA: Traditional birth attendant
